# Inflammation-associated changes in lipid composition and the organization of the erythrocyte membrane

**DOI:** 10.1016/j.bbacli.2016.03.007

**Published:** 2016-04-03

**Authors:** Sip Dinkla, Lucas T. van Eijk, Beate Fuchs, Jürgen Schiller, Irma Joosten, Roland Brock, Peter Pickkers, Giel J.C.G.M. Bosman

**Affiliations:** a Department of Biochemistry, Radboud University Medical Center, Radboud Institute for Molecular Life Sciences, Nijmegen, The Netherlands; b Department of Laboratory Medicine — Laboratory of Medical Immunology, Radboud University Medical Center, Nijmegen, The Netherlands; c Department of Intensive Care Medicine, Radboud University Medical Center, Nijmegen, The Netherlands; d University of Leipzig, Medical Faculty, Institute of Medical Physics and Biophysics, Leipzig, Germany

**Keywords:** Erythrocyte, Inflammation, Sepsis, Lipid metabolism, Lysophosphatidylcholine, Phosphatidylserine

## Abstract

**Background:**

Reduced erythrocyte survival and deformability may contribute to the so-called anemia of inflammation observed in septic patients. Erythrocyte structure and function are affected by both the membrane lipid composition and the organization. We therefore aimed to determine whether these parameters are affected during systemic inflammation.

**Methods:**

A sensitive matrix-assisted laser desorption and ionization time-of-flight mass spectrometric method was used to investigate the effect of plasma components of 10 patients with septic shock and of 10 healthy volunteers subjected to experimental endotoxemia on erythrocyte membrane lipid composition.

**Results:**

Incubation of erythrocytes from healthy control donors with plasma from patients with septic shock resulted in membrane phosphatidylcholine hydrolysis into lysophosphatidylcholine (LPC). Plasma from volunteers undergoing experimental human endotoxemia did not induce LPC formation. The secretory phospholipase A_2_ IIA concentration was enhanced up to 200-fold in plasma of septic patients and plasma from endotoxin-treated subjects, but did not correlate with the ability of these plasmas to generate LPC. Erythrocyte phosphatidylserine exposure increased up to two-fold during experimental endotoxemia.

**Conclusions:**

Erythrocyte membrane lipid remodeling as reflected by LPC formation and/or PS exposure occurs during systemic inflammation in a secretory phospholipase A_2_ IIA-independent manner.

**General significance:**

Sepsis-associated inflammation induces a lipid remodeling of the erythrocyte membrane that is likely to affect erythrocyte function and survival, and that is not fully mimicked by experimental endotoxemia.

## Highlights

• Erythrocyte membrane lipid remodeling occurs during systemic inflammation.
• Erythrocyte phosphatidylcholine hydrolysis during sepsis does not rely on SPLA_2_ IIA.
• Experimental endotoxemia does not fully mimic the effects of sepsis on erythrocytes.

## 1 Introduction

In patients with inflammation, anemia is associated with poor patient outcome. Next to changes in iron homeostasis and defective erythropoiesis [1], [2], reduced erythrocyte lifespan contributes to “anemia of inflammation” [3], [4], [5]. This condition is common in patients suffering from sepsis [6]. Erythrocyte shape, deformability, and aggregability were shown to be altered in these patients [7], [8]. Such structural and functional changes may contribute to the microcirculatory alterations that are linked to untimely erythrocyte removal in the spleen [9] and to a poor outcome [8].

Erythrocyte structure and function depend on plasma membrane lipid composition [10], [11]. Sepsis-associated alterations in membrane lipid composition may contribute to alterations in erythrocyte function. Indeed, incubation of erythrocytes from healthy volunteers with plasma of septic patients has been shown to induce phosphatidylserine (PS) exposure and membrane ceramide formation [12], both with functional consequences [10], [13].

Phospholipids constitute the majority of the erythrocyte membrane lipids, with the glycerophospholipid (GPL) phosphatidylcholine (PC) and the sphingolipid sphingomyelin (SM) dominating the outer membrane of the lipid bilayer [10], [11]. Secretory phospholipase A_2_ (sPLA_2_) and sphingomyelinase (SMase) catalyze the hydrolysis of GPLs into lysophospholipids (LPLs) and free fatty acids, and SM into ceramide and choline, respectively. The activity of both lipases is enhanced in the plasma of patients with sepsis [14], [15], and the lipids they generate are involved in the pathology of inflammation [16]. *In vitro*, erythrocyte deformability and survival are negatively influenced by sPLA_2_ and SMase, and by the incorporation of their products into the membrane [9], [13], [17], [18].

The aim of the current study was to investigate the involvement of lipase activity in the erythrocyte-related pathophysiology during systemic inflammation in patients with sepsis and in experimental human endotoxemia. Using sensitive matrix-assisted laser desorption and ionization time-of-flight (MALDI-TOF) mass spectrometry (MS), we investigated the lipid composition of erythrocytes after incubation with the plasma of patients suffering from septic shock and with plasma of subjects undergoing experimental endotoxemia. A mechanistic explanation for the observed changes was explored by measuring plasma sPLA_2_ IIA levels and erythrocyte PS exposure.

## 2 Material and methods

### 2.1 Septic patients

Anticoagulated blood (4 mL) was collected from 10 septic shock patients who resided in the department of Intensive Care Medicine of the Radboud University Medical Center, Nijmegen, the Netherlands, and from ten healthy volunteers. Septic shock was defined as having two or more systemic inflammatory response syndrome criteria [19], in combination with a proven or suspected infection and the need for vasopressor therapy following adequate fluid resuscitation. The study was carried out in accordance with the applicable rules on review by ethics committees and informed consent. Blood from all patients was drawn within 24 h after starting vasopressor therapy. Plasma, erythrocytes and erythrocyte membrane fractions were obtained by differential centrifugation.

### 2.2 Human endotoxemia trial

This study was part of a larger endotoxemia trial (*clinicaltrials.gov* *identifier*: *NCT01349699*) [20]. Our analyses were performed on data of the ten placebo-LPS-treated subjects only. The trial was approved by the local ethics committee, and carried out according to GCP standards and the declaration of Helsinki. A detailed protocol of the human endotoxemia trial was previously described [20]. Blood was collected at various time points after the injection of purified endotoxin (LPS) prepared from *E. coli* O:113 (Clinical Center Reference Endotoxin, National Institute of Health (NIH), Bethesda, MD, USA) [20].

### 2.3 sPLA_2_ IIA and cytokine measurements

sPLA_2_ IIA concentrations were determined by ELISA (Cayman Chemical, Ann Arbor, MI, USA). Tumor necrosis factor (TNF)-α and interleukin-6 (IL-6) were measured by Luminex (Bio-Plex cytokine assay, Bio-Rad, Hercules, CA, USA).

### 2.4 Erythrocyte isolation from blood group O, Rhesus-negative donors

EDTA-anti-coagulated blood was collected from several blood group O, Rhesus-negative volunteers, and erythrocytes were isolated using Ficoll (GE Healthcare, Waukesha WI, USA) density centrifugation.

### 2.5 Plasma incubation

Blood group O, Rhesus-negative erythrocytes were incubated in the plasma of patients or healthy volunteers at 10% hematocrit in a final volume of 500 μL, for 20 h at 37 °C with gentle agitation. Erythrocytes incubated with calcium-containing (2.5 mM CaCl_2_) Ringer with or without bee venom sPLA_2_ type III (Cayman Chemical, Ann Arbor, MI, USA) served as positive and negative controls. Absorption at 415 nm was determined to assess the extent of hemolysis.

### 2.6 Flow cytometry

Erythrocytes were probed for PS exposure with Annexin V-FLUOS (Roche, Basel, Switzerland). Flow cytometry was performed as described previously [21].

### 2.7 MALDI-TOF MS detection of membrane lipids

Erythrocytes (50 μL) were lysed and the membrane fractions were washed. Membrane lipids were extracted and analyzed by MALDI-TOF MS [22], [23], [24], [25], [26].

### 2.8 Thin layer chromatography (TLC)

Lipids were separated using high performance TLC silica gel 60 plates, lipids were visualized and MALDI mass spectra were recorded directly from the TLC plate as described previously [26], [27].

### 2.9 Statistical analysis

Differences in the percentages of total membrane lysophosphatidylcholine (LPC) between two groups were determined using Fisher's exact test. A repeated measures one-way ANOVA was used in combination with Tukey's post-test to assess changes in erythrocyte PS exposure over time. Differences between two groups of continuous data were determined using the Mann–Whitney U test. The relation between two parameters was assessed by Pearson correlation. Reported values are two-sided, and a *P*-value of < 0.05 was used for statistical significance.

Additional details of the methods used are given in the Supplemental Information.

## 3 Results

### 3.1 Demographic characteristics

Characteristics of the septic patients are provided in Table 1, and characteristics of the volunteers who participated in the endotoxemia trial (trial subjects) and of the control donors that served as controls for the septic patient group are presented in Table 2.

### 3.2 Enhanced LPC generation in erythrocytes incubated with septic patient plasma

**Fig. 1:**
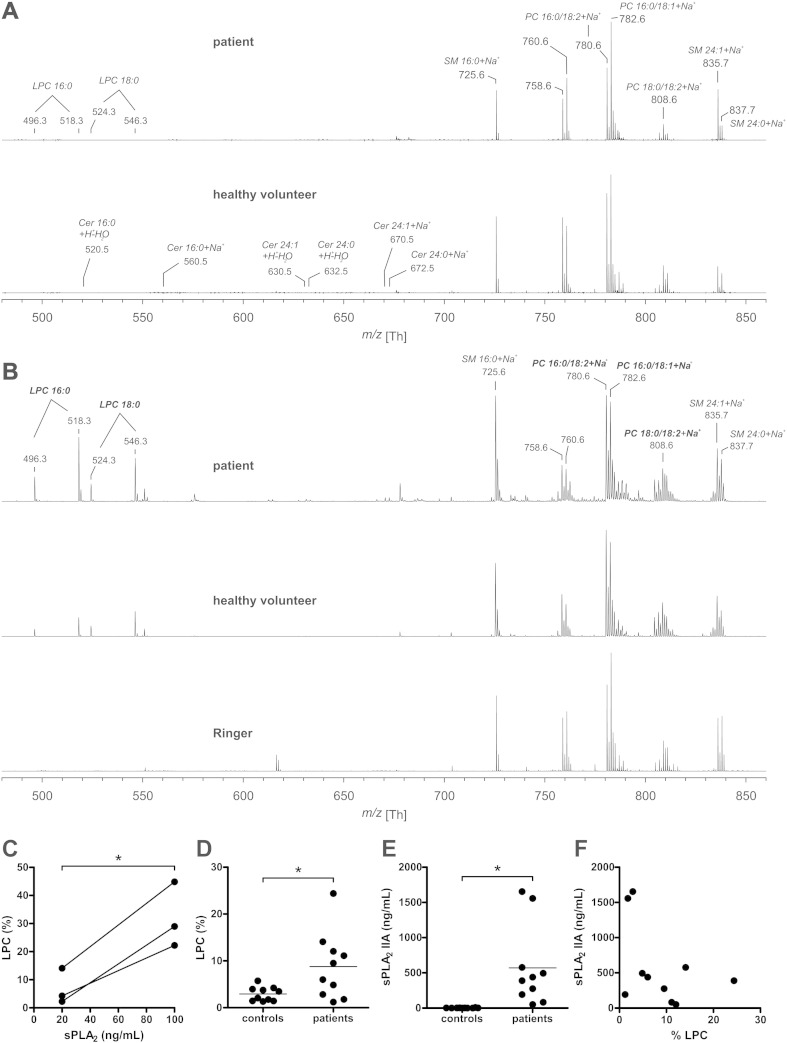
Septic patient plasma-induced LPC formation in the erythrocyte membrane. Five representative examples of positive-ion MALDI-TOF MS spectra of membrane lipid extracts of erythrocytes from septic patients, healthy controls (A), or control donor erythrocytes incubated overnight at 37 °C with i) plasma from septic patients, ii) plasma from healthy controls, and iii) Ringer solution (B). All peaks are labeled according to their mass-to-charge (*m*/*z*) ratios and assignments of the most prominent peaks are given directly in the figure. Also, the *m*/*z* ratios of LPC and ceramide are indicated in panel A and panel B, respectively. The percentage of hydrolyzed PC (LPC) was determined by comparing the proton and sodium adducts of LPC 16:0 (*m*/*z* 496.3 and 518.3), to the combined pool of LPC 16:0 and PC 16:0/18:2 (*m*/*z* 758.6 and 780.6). LPC formation after incubation of erythrocytes from three different donors with Ringer containing 20 and 100 ng/mL sPLA_2_ (C). LPC formation after overnight incubation with plasma from 10 septic patients and 10 healthy controls (D). The human sPLA_2_ IIA concentration in all plasma samples was determined using an ELISA (E). The sPLA_2_ IIA concentrations measured in patients did not correlate (*r* = − 0.44, *P* = 0.22) to the observed LPC percentage (F). Means are shown; *: *P* < 0.05. Abbreviations: Cer = Ceramide, LPC = lysophosphatidylcholine, PC = phosphatidylcholine and SM = sphingomyelin.

Lipid analysis of erythrocytes from patients with septic shock and from control donors did not reveal the presence of lysolipids such as ceramide and LPC (Fig. 1A). These observations may yield a skewed picture due to the loss of compromised cells by lysis or phagocytosis in the circulation. Therefore, incubation of erythrocytes with plasma of septic patients was used to investigate sepsis-associated lipid remodeling in the circulation. The number of PS-exposing erythrocytes was within the normal range observed *in vivo* [21] after incubation with patient (0.39% ± 0.11) and with control donor (0.43% ± 0.07) plasma samples. Incubation with septic patient plasma, and to a much lesser extent with control donor plasma, caused the formation of LPC (Fig. 1B). Ceramide and reactive oxygen species-generated LPLs were not observed (Fig. 1B), even though they are readily detectable by MS [28]. The observed LPC percentages were within the same range as observed after incubation with sPLA_2_ (Fig. 1C). LPC formation was significantly enhanced in erythrocytes treated with plasma from septic patients, but not in erythrocytes treated with plasma from control donors (Fig. 1D). The percentage of LPC in the total plasma PC pool was much lower for the septic patients (0.63 ± 0.35, n = 3) than for the control donors (4.91 ± 0.49, n = 3), making the incorporation of LPC from the plasma into the erythrocyte membrane an unlikely cause of the increase observed after incubation with patient plasma.

### 3.3 Enhanced LPC generation does not correlate with the sPLA_2_ IIA plasma concentration

The enzyme sPLA_2_ type IIA is the only member of the sPLA2 family that is increased in the plasma of septic patients [29]. Indeed, all our septic patients had enhanced plasma sPLA_2_ IIA concentrations (50.2–1654.0 ng/mL) as compared to the control donors (1.3–9.3 ng/mL) (Fig. 1E), corroborating earlier observations [15], [29]. However, there was no correlation between the observed degree of LPC formation and the sPLA_2_ IIA concentration (Fig. 1F). Also, no correlations were observed between the percentage of LPC and the sPLA_2_ IIA concentration with the patient parameters sex, age, weight, length, focus of infection, APACHE II, temperature, mean arterial pressure, heart rate, fluid balance, Glasgow coma scale, PaO_2_, FiO_2_, thrombocytes, bilirubin, creatinine or leukocytes (data not shown).

### 3.4 LPC generation and sPLA_2_ IIA plasma concentration in human endotoxemia

**Fig. 2:**
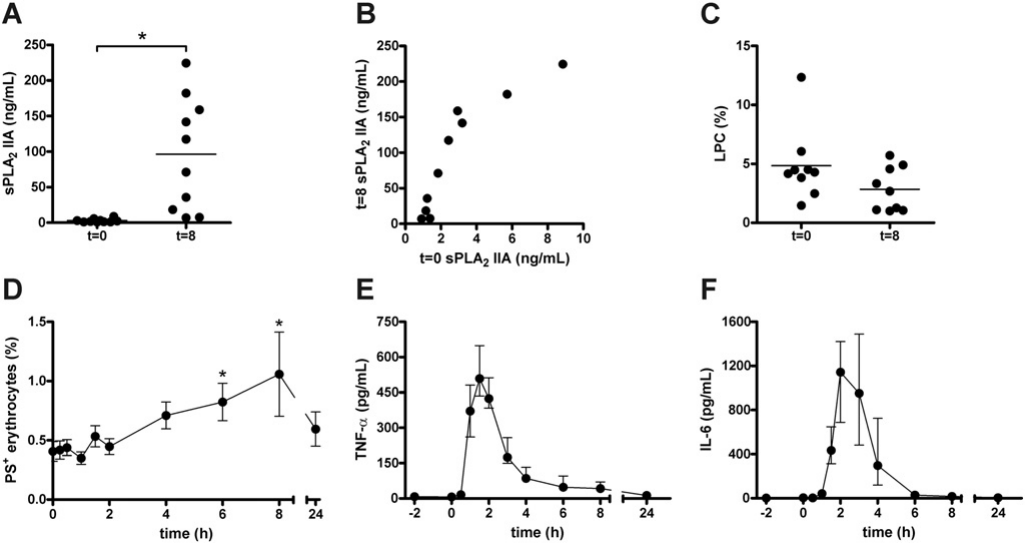
Erythrocyte lipid remodeling during human experimental endotoxemia-induced inflammation. Allogeneic erythrocytes were incubated overnight at 37 °C with plasma obtained from 10 endotoxemia trial subjects just prior to (t = 0) and 8 h after (t = 8) the administration of 2 ng/kg clinical grade LPS. The sPLA_2_ IIA concentration in all plasma samples was determined using an ELISA (A). The sPLA_2_ IIA concentrations at t = 0 were correlated to the concentrations at t = 8 (*r* = 0.89, *P* < 0.001) (B). The percentage of hydrolyzed PC (LPC) was determined by positive-ion MALDI-TOF MS, by comparing the intensities of the proton and sodium adducts of LPC 16:0 (*m*/*z* 496.3 and 518.3) with the combined pool of LPC 16:0 and PC 16:0/18:2 (*m*/*z* 758.6 and 780.6) (C). Of the same 10 trial subjects, the percentage of PS-exposing erythrocytes was determined by flow cytometry detection of Annexin V-FLUOS staining (D), and TNF-α (E) and IL-6 (F) plasma concentrations were assessed by Luminex. In panels A, C and D means are shown; *: *P* < 0.05; error bars in panel D represent SEM. In panels E and F median values are shown, and the error bars represent the inter-quartile range.

In order to overcome the heterogeneity in origin of infection, bacteriology and progression of sepsis [30], we investigated the effect of sepsis on erythrocyte lipid composition using a human endotoxemia model [20]. After LPS infusion, the sPLA_2_ IIA concentrations increased in almost all trial subjects (Fig. 2A). A strong correlation (*r* = 0.89, *P* < 0.001) was observed between sPLA_2_ IIA levels before and 8 h after LPS infusion (Fig. 2B). Furthermore, the sPLA_2_ IIA concentration at t = 0 correlated (*r* = 0.68, *P* = 0.032) with the rise in body temperature after LPS infusion. However, in contrast to that of septic patients, the plasma of the trial subjects did not induce LPC formation in erythrocytes (Fig. 2C), even though its sPLA2 IIA levels overlapped with those of septic patients (cf. Figs. 1D and 2A). This corroborates our finding that, in septic patients, sPLA_2_ IIA concentration is not related with the LPC formation (Fig. 1E).

### 3.5 Erythrocyte PS exposure is induced during the initial phase of human endotoxemia

*In vivo*, LPC is rapidly converted into lysophosphatidic acid (LPA), which may induce the exposure of the removal signal PS in erythrocytes [31]. In trial subjects, the number of PS-exposing erythrocytes increased strongly at 6 and 8 h after LPS infusion, to return to baseline level at 24 h (Fig. 2D). This increase in PS-exposing erythrocytes was observed after the onset of inflammation, as indicated by the TNF-α and IL-6 plasma levels (Fig. 2E, F). There was a strong positive correlation (*r* = 0.91, *P* = 0.002) between erythrocyte PS exposure before and at 6 h after LPS infusion.

### 3.6 The observed lipase activity selectively targets PC in the erythrocyte

**Fig. 3:**
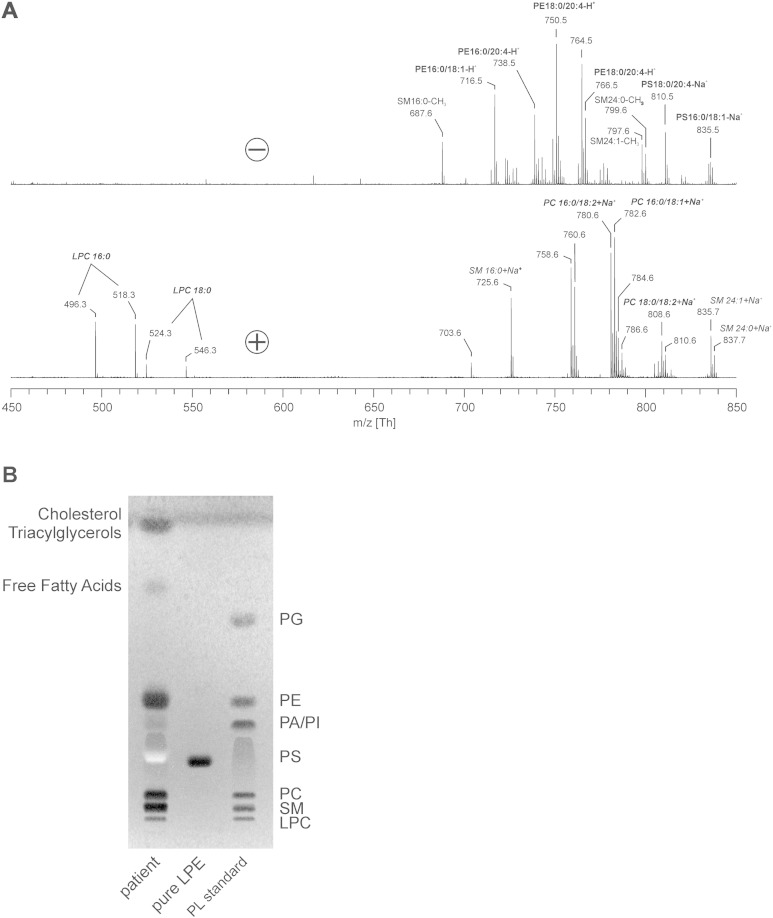
Detection of lysophospholipids. Allogeneic erythrocytes were incubated overnight at 37 °C with plasma of a septic patient. Both positive and negative-ion MALDI-TOF MS of isolated membrane lipids were performed (A). All peaks are labeled according to their *m*/*z* ratios. Note that there are intense signals of LPC, but no signals of other LPL species. TLC was performed using the identical lipid extract (B) to verify the absence of LPL other than LPC. Lipid standards were included to delineate the different lipid species. The pale white band in the TLC is caused by the presence of small amounts of hemoglobin that could not be completely removed during the extraction process [33]. Abbreviations: LPC = lysophosphatidylcholine, LPE = lysophosphatidylethanolamine, PA = phosphatidic acid, PC = phosphatidylcholine, PE = phosphatidylethanolamine, PG = phosphatidylglycerol, PI = phosphatidylinositol, PL = phospholipid, PS = phosphatidylserine and SM = sphingomyelin.

sPLA_2_ phospholipases may not only hydrolyze PC, but also other glycerophospholipids such as PS and phosphatidylethanolamine (PE) [32]. Incubation of control donor erythrocytes with septic patient plasma generated high levels of LPC, but no other LPLs such as lysoPE (LPE), lysoPS, or lysophosphatidylinositol (Fig. 3A, B). Also, incubation in Ringer that had been spiked with recombinant sPLA_2_ did not generate any LPLs other than LPC (data not shown). Most likely the plasma phospholipases are unable to attack other phospholipids *in situ* because of their location in the inner leaflet of the erythrocyte membrane.

## 4 Discussion

sPLA_2_ phospholipases are the principal catalysts of the hydrolysis of GPLs into LPLs and fatty acids, while SMases catalyze the hydrolysis of SM into ceramide and phosphorylcholine. Both activities are enhanced in patients with severe sepsis [14], [15], and LPLs and ceramide have been shown to play a role in the pathology of various inflammatory diseases [16]. sPLA_2_ has attracted attention as a therapeutic target in atherosclerosis [32], [34]. Here, we report that the plasma of patients with severe sepsis triggers erythrocyte membrane lipid remodeling, as shown by enhanced LPC formation. This LPC formation was similar to that observed after incubation with sPLA2 (Fig. 1). sPLA_2_ IIA is the only sPLA_2_ family member of which the secretion is significantly promoted during sepsis [29]. Indeed, the sPLA_2_ IIA concentration was strongly enhanced in our septic patient plasma samples. The lack of a statistically significant correlation between sPLA2 concentration and LPC formation is likely due to the involvement of other sPLA2 phospholipases [29]. Interestingly, we did not detect an increase in LPC generation using the plasma of subjects undergoing experimentally induced endotoxemia, even though inflammation and a subsequent rise in plasma sPLA_2_ IIA were observed. Thus, experimentally-induced acute endotoxemia may constitute a useful model for several aspects of sepsis, but does not fully mimic the effects of sepsis on erythrocytes. This could be due to the human endotoxemia model mimicking only the initial phase of septic shock [20]. The absence of LPC formation in the endotoxemia model suggests that additional factors, or the exposure of PS, direct sPLA_2_ IIA towards compromised erythrocytes.

sPLA_2_ IIA has been implicated in the activation of the inflammatory processes responsible for multiple organ failure in sepsis [30], [35], and its plasma level correlated with the rate of mortality [15], [36]. In the human endotoxemia trial subjects, we noticed that sPLA_2_ IIA status at baseline was predictive for the sPLA_2_ IIA response upon LPS infusion. This suggests that there is a pre-existing, inter-individual variability in the sPLA_2_ IIA response to systemic inflammation.

Our MS method enables both specific and sensitive detection of different ceramide species in membrane lipid preparations [37]. However, and in contrast to earlier findings [12], we did not observe ceramide after erythrocyte incubation with septic patient plasma. As we could detect ceramide after sphingomyelinase treatment *in vitro* [13] this discrepancy may be caused by patient heterogeneity in the stage and cause of sepsis.

The absence of LPLs other than LPC may be due to the inability of the responsible plasma lipases to attack their substrate GPLs, since the latter primarily reside in the inner leaflet of the plasma membrane [10], [11]. LPC acts as a substrate for lysophospholipase D and LPC acyltransferase, to produce the pro-inflammatory lipid mediators LPA and platelet-activating factor [38]. Enhanced activity of these enzymes might explain why LPC levels are reduced in the plasma of septic patients [39].

We observed a small increase in the percentage of PS-exposing erythrocytes in the trial subjects after LPS infusion. Since PS-exposing erythrocytes are rapidly removed from the circulation, the increase in PS-exposing erythrocytes observed in endotoxemia subjects presumably does not represent the actual number of erythrocytes that expose PS after LPS infusion. In addition, significant LPC build-up in the erythrocyte membrane can induce hemolysis [18], [40], alter morphology, induce microparticle release, and reduce deformability leading to splenic retention *ex vivo* [9]. Both the removal and metabolism hypotheses may explain our inability to detect LPC in the erythrocytes from the septic patients. PS exposure and PC hydrolysis in the erythrocyte membrane may contribute to the reduced erythrocyte lifespan in “anemia of inflammation” [3], [4], [5]. This might also affect erythrocyte transfusion in septic patients, as blood bank erythrocytes are prone to expose PS [41]. We found that, in trial subjects, erythrocyte PS exposure prior to LPS infusion was predictive for the extent of erythrocyte PS exposure upon LPS-induced inflammation. This is in line with our previous finding that PS exposure on stored erythrocytes predicts their extent of PS exposure after the application of osmotic stress [21]. Therefore, determination of the PS exposure in erythrocyte concentrates might allow the selection of products for the transfusion of septic patients. Since blood bank erythrocytes were also found to be more susceptible for SMase activity, it would be interesting to assess whether this also applies to sPLA_2_s [13].

## 5 Conclusions

We here provide evidence for erythrocyte membrane lipid remodeling during systemic inflammation, as illustrated by enhanced LPC formation and PS exposure during sepsis. The enhanced LPC formation in erythrocytes could not be attributed to sPLA_2_ IIA alone, and was not observed in experimentally induced endotoxemia. As membrane lipid remodeling affects erythrocyte integrity, it is necessary to focus future research on elucidating the identity of the phospholipase(s) responsible, including erythrocyte-associated forms [42], [43]. Comparing the endotoxemia model with septic patients would be helpful in short-listing relevant candidates.

## Authorship and disclosures

SD performed the biochemical analyses, LvE and PP designed the clinical part of the investigation, recruited the patients and volunteer donors, obtained the blood samples and performed the clinical analyses, BF and JS performed the lipid analyses; SD, IJ, RB, PP and GB conceived and designed the study; all authors contributed to the preparation of the manuscript.

This research was funded by the German Research Council (DFG FU 771/1-3) and the Radboud University Medical Center, Nijmegen, The Netherlands.

The authors have no potential conflicts of interest.

## Transparency Document

Transparency document. Image 1

## Figures and Tables

**Table 1:** Demographic characteristics of septic patients.

Pt Nr.	Sex (M/F)	Age (years)	Diagnosis	APACHE II	Hb (mmol/L)	Transfusion history
1	V	54	Pneumosepsis	28	4.9	None
2	M	68	Abdominal sepsis after intestinal ischemia		5.1	Day before blood drawing: 3 thrombocyte transfusions 3 plasma transfusions Day of blood drawing: 2 thrombocyte transfusions 8 plasma transfusions 9 erythrocyte transfusions
3	V	81	Abdominal sepsis in ulcerative colitis	28	6.0	None
4	M	59	Infected hip prosthesis		4.9	Day before blood drawing: 1 erythrocyte transfusion Day of blood drawing: 1 erythrocyte transfusion
5	M	75	Cholangitis	23	8.6	Day before blood drawing: 2 thrombocyte transfusions 2 plasma transfusions Day of blood drawing: 1 thrombocyte transfusion
6	M	82	Abdominal sepsis after intestinal ischemia	23	7.5	Day before blood drawing: 3 erythrocyte transfusions
7	M	34	Pneumosepsis	14	7.8	None
8	M	84	Urosepsis	16	6.3	None
9	V	51	Pneumosepsis	27	5.9	None
10	V	65	Toxicoderma	23	6.6	None
Mean ± SD		66.3 ± 16.1		22.8 ± 5.3	6.4 ± 1.3	

**Table 2:** Characteristics of healthy subjects.

	Endotoxemia subjects	Healthy volunteers
N	10	10
Age (years ± SD)	22.7 ± 2.8	34.7 ± 14.8
Sex (M/F)	10/0	8/2
